# Integrative Analysis Reveals the Landscape of Hypoxia-Inducible Factor (HIF) Family Genes in Pan-Cancer

**DOI:** 10.1155/2020/8873104

**Published:** 2020-11-24

**Authors:** Qian-Kun Yang, Xue-Xin Wang, Yang Wang, Ni Ni

**Affiliations:** ^1^Department of Bone and Soft Tissue Surgery, Cancer Hospital of China Medical University, Liaoning Cancer Hospital and Institute, Shenyang 110042, Liaoning, China; ^2^Department of Burns and Trauma, The First Affiliated Hospital, Naval Military Medical University, Shanghai 200433, China; ^3^Laboratory of Aging Research and Cancer Drug Target, State Key Laboratory of Biotherapy, National Clinical Research Center for Geriatrics, West China Hospital, Sichuan University, Chengdu, China; ^4^Department of Urology, Cancer Hospital of China Medical University, Liaoning Cancer Hospital and Institute, Shenyang 110042, Liaoning, China

## Abstract

Inside the cancer microenvironment, reduced O_2_ concentration, termed as hypoxia, is a common phenotype and leads to cancer progression. However, little is known about how and when those HIF members are dysregulated in distinct cancers. Here, by integrating a full range of data of thousands of patients, we comprehensively analyzed the genetics, epigenetics, and transcriptomic level of HIF genes and further defined pathways triggered by disrupted hypoxia-inducible factors. We reveal the expression landscape of HIF family genes and further demonstrate that copy number variations underlie such dysregulation. Further analysis indicates that HIF genes associate with cancer hallmarks such as cell cycle and DNA damage response. Drug resistance analysis showed that HIF globally impacts drug effectiveness such as docetaxel. In summary, the overall analysis reveals the landscape of HIF genes in pan-cancer and may assist mechanism research about hypoxia.

## 1. Introduction

For cells to adapt to hypoxia, they must be able to sense changes in oxygen and respond to them [1, 2]. Under such a scenario, hypoxia-inducible factors (HIFs) mediate adaptive physiological responses to hypoxia [3–5]. The HIF transcriptional complex transcriptionally activates molecules modulating oxygen homeostasis and metabolic activation [6, 7]. Eight members, including hypoxia-inducible factor 1 (HIF-1*α*, also termed as HIF1A) [8], aryl hydrocarbon receptor nuclear translocator (HIF-1*β*, also termed as ARNT) [9], endothelial PAS domain protein 1 (HIF-2*α*, also termed as EPAS1) [10], aryl hydrocarbon receptor nuclear translocator 2 (HIF-2*β*, also termed as ARNT2) [11], hypoxia-inducible factor 3 (HIF-3*α*, also termed as HIF3A) [12], and aryl hydrocarbon receptor nuclear translocator 3 (HIF-3*β*, also termed as ARNT3) [13], formed this transcriptional complex [14]. In normal cells, these members maintain the balance of oxygen metabolism and adaptive physiological responses to hypoxia [3].

Particularly in cancer cells, the HIF pathway is widely accepted to be enhanced or upregulated [15]. Inside the tumor, reduced O_2_ availability is a common phenotype and leads to cancer hallmarks such as angiogenesis, metabolic reprogramming, and epithelial-mesenchymal transition [15]. Yet, little is known about how and when these HIF members are dysregulated. Copy number variations (CNVs), genetic mutation, and promoter methylation will all exert large effects on gene expression; however, how these factors contribute to HIF enhancement is unknown. We hence seek to address the origin of such upregulation and quantify the most enriched pathways linked with such upregulation. Defining enriched pathways of hypoxia might help improve our understanding of how HIFs shape cancer cells and offer the promise of developing targeting drugs.

Here, by integrating multiple data of thousands of patients, we aim to explore how cancer cells reprogram the HIF transcriptional program. We comprehensively analyzed the genetics, epigenetics, and transcriptomic level of HIF genes and further defined pathways triggered by disrupted hypoxia-inducible factors. This proposed work will elucidate key pathways in response to hypoxia that may underlie critical steps in carcinogenesis, genetic instability, tumor progression, and resistance to cancer therapies. Identification of strategies to prevent or reverse these pathways may provide the basis for new approaches to cancer prevention and therapy.

## 2. Methods

### 2.1. Data Source

As for the gene expression analysis, methylation analysis, single nucleotide variation analysis, copy number variation analysis, and pathway enrichment analysis, we downloaded data from The Cancer Genome Atlas (TCGA, https://portal.gdc.cancer.gov/). As for the normal tissue gene expression analysis, we downloaded data from GTEx (https://www.gtexportal.org/home/datasets). As for drug response analysis, we downloaded data from GDSC (http://www.cancerrxgene.org/).

### 2.2. Gene Expression Analysis

As for the differential gene expression analysis, we used mRNA count data of cancer samples and the matched normal samples. We used Deseq2 (http://bioconductor.org/packages/devel/bioc/vignettes/DESeq2/inst/doc/DESeq2.html) to perform the differential gene expression analysis. We set the cutoff as 2 and 0.05 for the fold change and FDR. We used *R* to perform the Spearman correlation analysis.

### 2.3. Methylation Analysis and Single Nucleotide Variation Analysis

As for the differential methylation analysis, we used methylation probe data of cancer samples and the normal samples. We used Student's *t* test to perform the differential methylation analysis. We set the cutoff as 0.05 for the FDR. The mRNA expression and methylation data were matched according to the barcode of each sample. We used *R* to perform the Spearman correlation analysis between methylation and mRNA expression. We used maftools (https://github.com/PoisonAlien/maftools) to summarize the single nucleotide variation events (driver mutation).

### 2.4. Copy Number Variation Analysis

We divided all CNV events into heterozygous and homozygous events, which means the CNV occurs on only one or two chromosomes. We use GISTICS (ftp://ftp.broadinstitute.org/genepattern/modules_public_server_doc/GISTIC2.pdf) to analyze the CPV events. Only genes with >5% CNV in cancers were shown as a corresponding point on the figure. We used *R* to perform the Spearman correlation analysis between CNV and mRNA expression.

### 2.5. Pathway Enrichment Analysis

We calculated the pathway score according to the reverse-phase protein array data in TCGA. RPPA data are centered on the median, and each component is normalized by the STD of all patients to obtain relative protein levels. Gene expression was divided into two groups according to the median value (high and low), and Student's *t* test was used to determine the difference in the pathway activity score (PAS). The heat map shows genes that are functional (suppressed or activated) in at least 5 cancer types. The pathway_a (red) represents the percentage of cancers that are activated by specific genes and is inhibited similarly to the pathway_a (blue).

### 2.6. Drug Sensitivity Analysis

To analyze the correlation between gene expression and drug sensitivity, we downloaded drug dose-response curve (AUC) values and regions under the gene expression profile for all cancer cell lines. Fisher's *Z* transform was used to normalize the transcription level and the Pearson correlation coefficient of the area under the curve. The two-tailed distribution corrected by Bonferroni had a family error rate of *z*-scores less than 0.025. The Spearman correlation coefficient of the agent target was compared with the same number of drug-gene pairs computed by random sampling correlation.

## 3. Results

### 3.1. Hypoxia-Inducible Factors Are Dysfunctional across Cancers Which Leads to Worse Patient Outcomes

**Figure 1:**
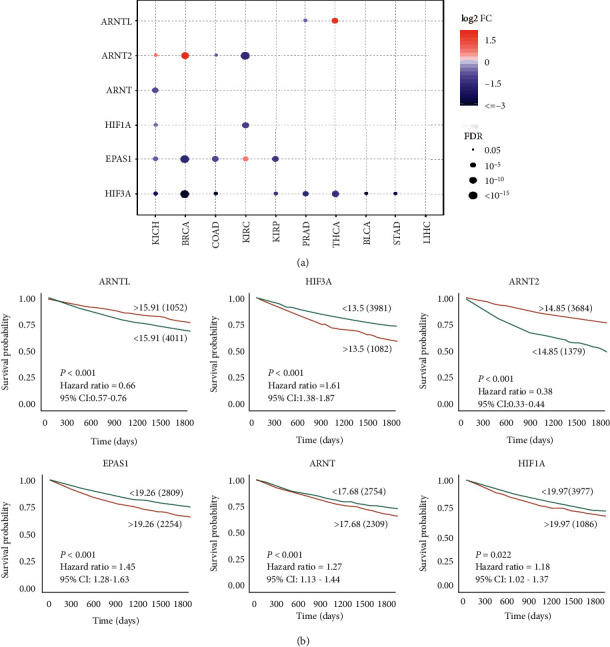
Hypoxia-inducible factor family genes are widely dysregulated in human cancers. (a) The HIF genes are downregulated in a wide range of cancers. (b) Abnormal HIF gene expression associates with favorable or unfavorable patient survival in pan-cancer. FC: fold change, FDR: false discovery rate, THCA: thyroid carcinoma, KIRP: kidney renal papillary cell carcinoma, LIHC: liver hepatocellular carcinoma, STAD: stomach adenocarcinoma, BRCA: breast invasive carcinoma, COAD: colon adenocarcinoma, UCEC: uterine corpus endometrial carcinoma, BLCA: bladder urothelial carcinoma, KIRC: kidney renal clear cell carcinoma, KICH: kidney chromophobe, PRAD: prostate adenocarcinoma.

We used the differential gene analysis of hypoxia-inducible factor family members according to the raw count of these HIF genes from ten cancer types downloaded from The Cancer Genome Atlas. We selected cancers withed matched normal samples including THCA, KIRP, LIHC, STAD, BRCA, COAD, UCEC, BLCA, KIRC, KICH, and PRAD. We found that the majority of the detected HIF genes are significantly changed (Figure 1). Among them, HIF3A and EPAS1 are significantly downregulated, whereas ATNTL and ARNT2 show minimal expression changes in most cancer types (Figure 1). We further found that HIF family gene expression varied in cancer subtypes (Figure S1). Especially in breast cancer, all HIF genes are differentially expressed in each subtype. In addition, we examined whether HIF gene expressions impact the overall survival of cancer patients (Figure 1). Our result demonstrates that all HIFs had either favorable prognostic or unfavorable prognostic impacts on overall survival. Nevertheless, the prognostic power varied by HIF factor types. Some HIFs, such as HIF1A, ARNT, EPAS1, and HIF3A, were linked with unfavorable outcomes for pan-cancer, while two HIFs including ARNTL and ARNT2 were associated with favorable outcomes in pan-cancer (Figure 1).

### 3.2. Heterozygous Amplification Contributes to Upregulated Hypoxia-Inducible Factors

**Figure 2:**
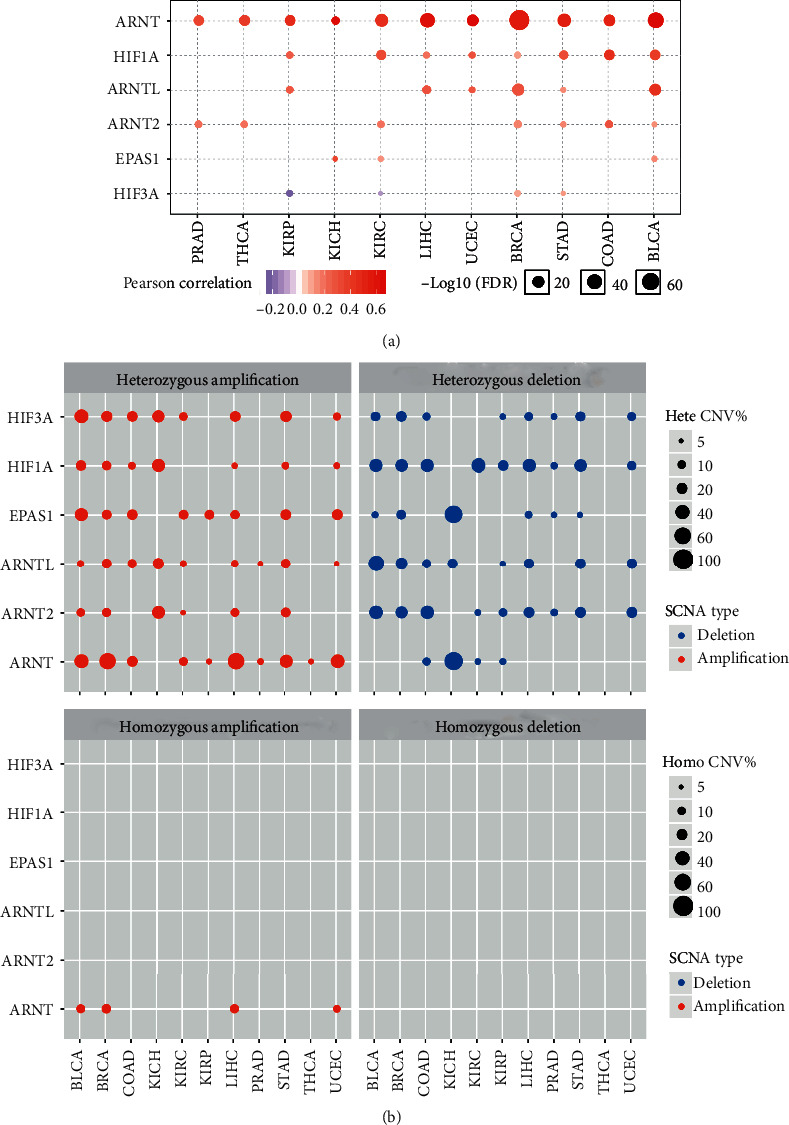
Copy number variations contribute to abnormal expression of hypoxia-inducible factors. (a) Copy number variations associate with HIF gene expression in cancers. (b) Copy number variation of the HIF gene includes heterozygous amplification and homozygous amplification. CNV: copy number variation, Hete: heterozygous, Homo: homozygous.

We also sought to explore the impact of copy number variations (CNVs) on HIF gene expression. We first separated all CNV events into heterozygous or homozygous copy number variations. Heterozygous amplification/deletion was mainly correlated with transcriptional alterations such as upregulated HIFs (e.g., HIF3A, ARNT, and ARNTL). Downregulated HIFs (e.g., HIF1A and ARNT2) were associated with decreased copy numbers (Figure 2 and Figure S2). However, we further examined the effect of homozygous amplification or deletion on mRNA level expression. To our surprise, only homozygous amplification of the ARNT gene associates with its upregulation in only 4 cancers (BLCA, BRCA, LIHC, and UCEC). This indicates that heterozygous copy number variations underlie the transcriptional changes of HIFs. To confirm our result, we perform the correlation analysis and observe the positive correlation between CNVs and mRNA expression in most HIF genes across cancers.

### 3.3. Mutation Profile of Hypoxia-Inducible Factors

**Figure 3:**
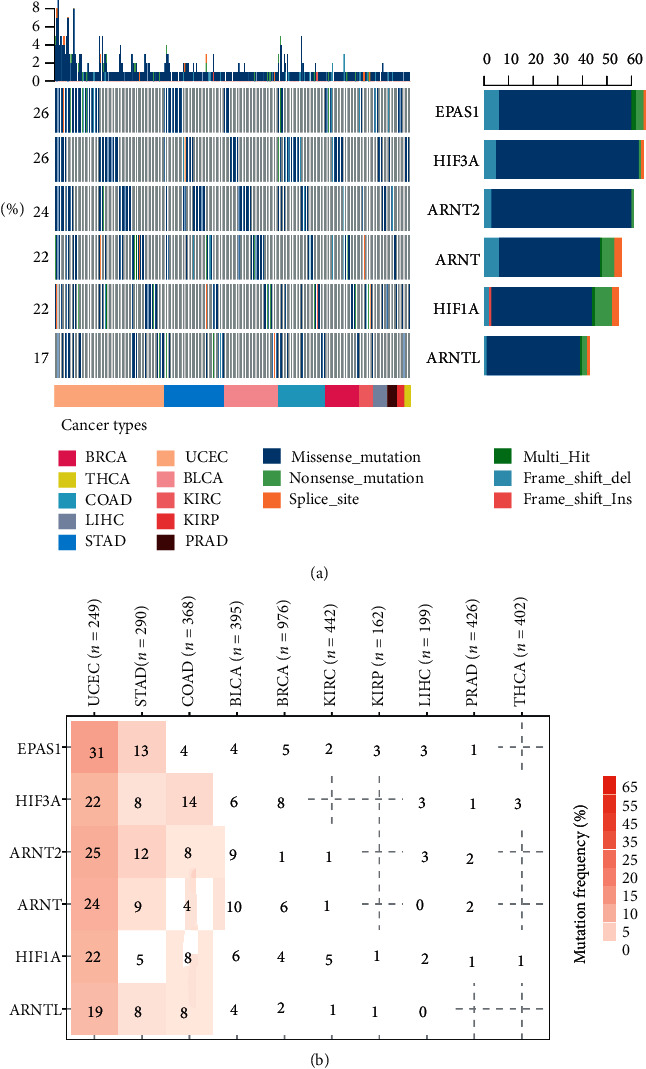
Mutations can also lead to HIF family dysfunction in cancers. (a) Frequency of HIF gene mutation. (b) A summary of the variation of each sample.

Based on the somatic mutation data from The Cancer Genome Atlas, mutations of HIFs widely exist (Figure 3). Even for the lowest-ranked HIFs like ARNTL, the mutation frequencies were around 17%. EPAS2 and HIF3A were mutated in ∼26% of samples. Most HIF mutations distribute in cancers with high mutation loads such as UCEC and STAD (Figure 3(a)). For example, especially in UCEC, 31% of patients show EPAS1 mutation (Figure 3(a)). Among all mutations, missense mutations predominate, and most of mutations are C to G (Figure S3). To sum up, frequent mutations of HIFs also contribute to the abnormal profile of hypoxia factors in cancers.

### 3.4. Demethylation Contributes to the Abnormal Hypoxia-Inducible Factors

**Figure 4:**
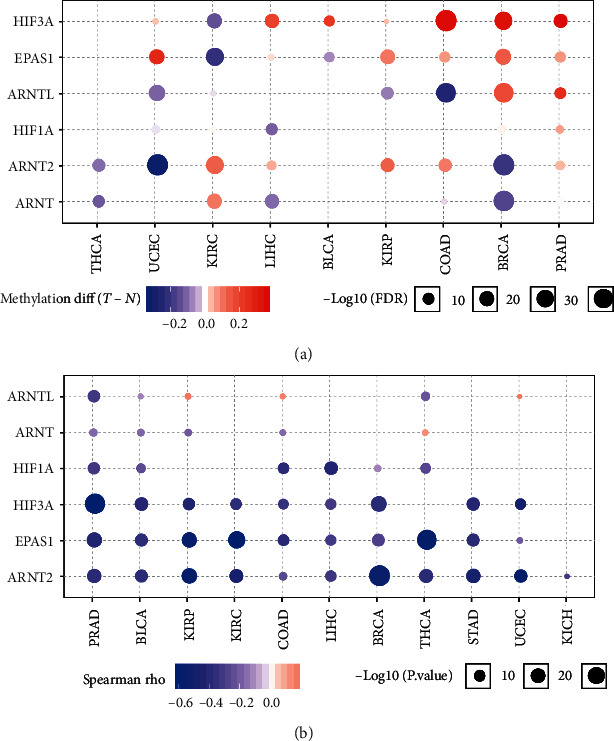
The HIF family is epigenetically methylated. (a) Differential methylation of the HIF genes in human cancers. (b) Correlation between promoter methylation and HIF gene expression.

Since the methylation of the gene promoters can induce the downregulation of genes, we downloaded the promoter methylation of HIF genes in different cancer types. We found the abnormal methylation of the hypoxia genes in multiple cancer types, especially in BRCA, PRAD, and LIHC (Figure 4(a)). Further, we utilized the Spearman correlation analysis to compute the correlation between the gene expression and methylation level of HIF genes across cancer types and observed that the methylation level of HIF1A, ARNT, EPAS1, ARNT2, HIF3A, and ARNTL are significantly negatively correlated with their expression level, respectively (Figure 4(b)), which may partially explain the abnormal expression of these HIF genes in cancer samples.

### 3.5. Abnormal Hypoxia-Inducible Factors Associates with Key Cancer Hallmarks

**Figure 5:**
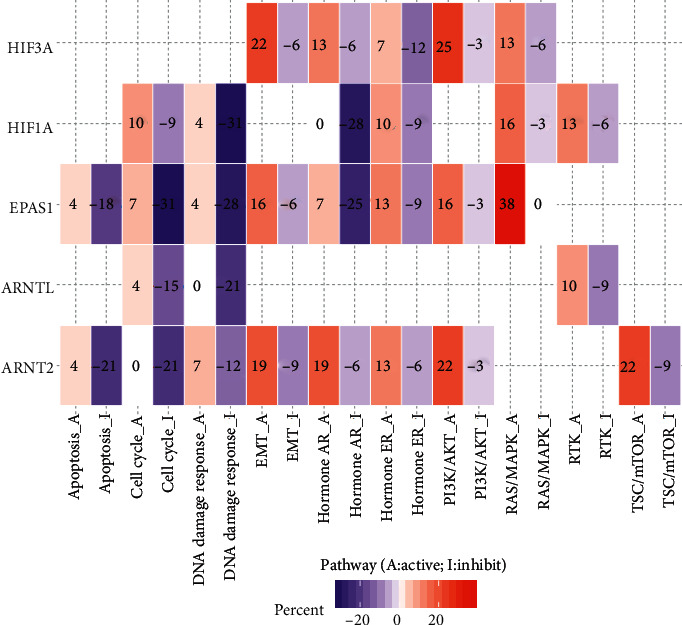
The HIF genes are widely associated with the hallmark cancer pathways. The heat map with function (inhibition or activation) in at least 5 cancer types. Pathway_a represents the potential activation of this pathway, and pathway_i represents the potential inhibition of this pathway.

We next explore the HIF-related cancer hallmark pathways, including apoptosis, cell cycle, DNA damage response, EMT, hormone AR, hormone ER, PI3K/AKT, RAS/MAPK, RTK, and TSC/mTOR. We found that upregulated HIF1A, ARNT, EPAS1, ARNT2, HIF3A, and ARNTL associates with the EMT signalling pathway (Figure 5 and Figure S4). Also, this observation is in consistency with previously reported results [16]. We also found previously unreported results. For example, HIF3A, which is reported to promote the cancer metastatic phenotype, was observed to be highly linked with PI3K pathway activation across multiple cancer types. In summary, our systematic analysis not only ensured the previously identified HIF-related pathways but also shed light on novel potential HIF-associated signalling pathways in human cancers.

### 3.6. Abnormal Hypoxia-Inducible Factors Associates with Drug Sensitivity

**Figure 6:**
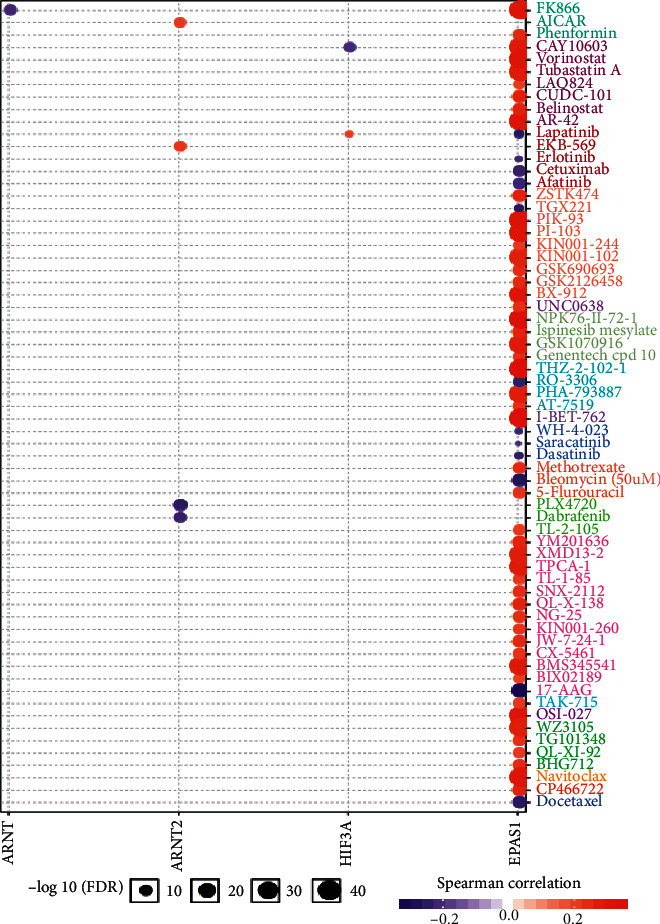
HIF family genes have a broad impact on drug sensitivity in cancer cell lines. Dots indicate the relationship between HIF gene expression and drug sensitivity. A positive correlation indicates that the high expression of this gene is resistant.

Many clinically actionable genes are targeted by anticancer drugs as identified in the GDSC project (Methods). Hypoxia in the tumor microenvironment is a common phenotype in cancers, and hence, targeting those hypoxia-related genes may help improve the survival of patients. To further assess how hypoxia impacts drug response, we computed the Spearman coefficient between drug sensitivity of 265 clinically used agents and mRNA expression of 6 HIFs across 1,080 cancer cell lines in GDSC. To our surprise, we observed no significant associations between HIF1A/ARNTL and drug response (Figure 6). In contrast, responses to 62 drugs are related to EPAS1 expression. For example, docetaxel, a chemotherapy medication used to treat many types of cancer, negatively correlates with EPAS1 expression. Taken together, these data demonstrate hypoxia genes, especially EPAS1, may affect drug sensitivity.

## 4. Discussion

Hypoxia in the tumor microenvironment can impact the cancer cell phenotypes [17]. Although previous studies have examined the role of HIF genes in some cancers, a system-level analysis is still lacking. HIF genes in cancers are highly context-dependent, making the role of HIFs complicated and changeable in distinct cancers, thus impeding the effective clinical utility of HIFs. Here, by integrating multiple data across cancer types, we seek to explore the landscape of hypoxia-inducible factors (HIFs) family genes.

First, we report the transcriptional landscape of HIF family genes in cancers. Hypoxia in the tumor microenvironment has been linked with cancer development, and hypoxia status is highly associated with effectiveness of anticancer drugs [18]. Our study presents the expression landscape of HIF genes, where most of them are dysregulated across cancer types. For example, hypoxia-inducible factor 3-alpha (HIF3A) is globally downregulated in cancers especially in breast cancer. This gene also shows a high rate (26%) of mutation in all samples. The pathway analysis of this gene shows its association with activation of EMT and PI3K, indicating this gene might serve as an important target in the clinical settings. Our system-level analysis represents a systematic computation of the widespread alterations of hypoxia-inducible factors in thousands of tumors.

Further, our research shows that HIF genes are associated with cancer hallmarks. Hypoxia contributes to the suppressive tumor microenvironment in many cancers by activating multiple pathways that allow tumor cells to escape from the innate and adaptive immune defenses [1, 19, 20]. To better understand how hypoxia impacts the pathways inside the tumor, we selected ten hallmark pathways. Our results demonstrated that most HIF genes negatively correlates with DNA damage response and cell cycle. This indicates that reduced O_2_ in the microenvironment may contribute to DNA damage. Our paper highlights the potentially linked signalling pathways of HIFs across a broad spectrum of cancers.

Finally, our study presents the interaction and correlation between the clinically actionable genes and HIFs, indicating that reprogramming the O_2_ concentration in the tumor microenvironment should be considered in cancer therapy [21]. In particular, endothelial PAS domain protein 1 (EPAS1) gene expression associates with responses to 62 drugs. This indicates that enhancing/blocking EPAS1 may serve as a promising combinational strategy in the clinical setting. However, we observe no significant associations between drug response and HIF1A/ARNTL gene expression, although they are core hypoxia regulators. In a nutshell, our paper provides more evidence that further efforts should be made to combine targeting hypoxia with existed drugs.

Our paper does have some limitations. These results should be validated in different patient cohorts. For example, future research should look at whether ARNTL can predict the outcome of patients. Second, The Cancer Genome Atlas does not document the synonymous mutation data. It is still unclear about the synonymous mutation of HIF genes and how they impact patients' prognosis.

In summary, our study reveals the expression landscape of HIF family genes and further addresses the origin, copy number variations, of such dysregulation. We also found that HIF genes associates with cancer hallmarks such as cell cycle and DNA damage response. Further drug resistance analysis showed that HIF globally impacts drug effectiveness such as docetaxel. These findings provide new insights into cancer hypoxia and unravel new mechanisms of HIF genes that may be further explored in the future.

## Data Availability

The dataset supporting the conclusions of this article is included within the article.

## Supplementary Materials

Supplementary Materials Figure S1: the expression of HIFs in normal tissues and cancer subtypes. (a) The mRNA expression of HIFs in normal tissues. (b) The expression of the HIF genes is different in cancer subtypes. Figure S2: copy number variation affects HIF gene expression. Copy number variant subtype of HIF gene in cancer. Hete Amp: heterozygous amplification; Hete Del: heterozygous deletion; Homo Amp: homozygous amplification; Homo Del: homozygous deletion; and no: no CNV. Figure S3: a review of HIF gene variants in human cancers. (a) Variant type, (b) variant classification, (c) single nucleotide variation, (d) variation per sample, (e) mutation classification, and (f) mutation of HIF gene between cancer types frequency. Figure S4: the HIF genes are widely associated with the hallmark cancer pathways in different cancer types. The pie plots represent the percentage of cancer samples that are correlated with HIF gene expression.
