# Supplementary figures and images for: Integrative Analysis Reveals the Landscape of Hypoxia-Inducible Factor (HIF) Family Genes in Pan-Cancer
Source: J Oncol. 2020 Nov 24;2020:8873104. doi: 10.1155/2020/8873104 (PMC7710422; doi:10.1155/2020/8873104)

A

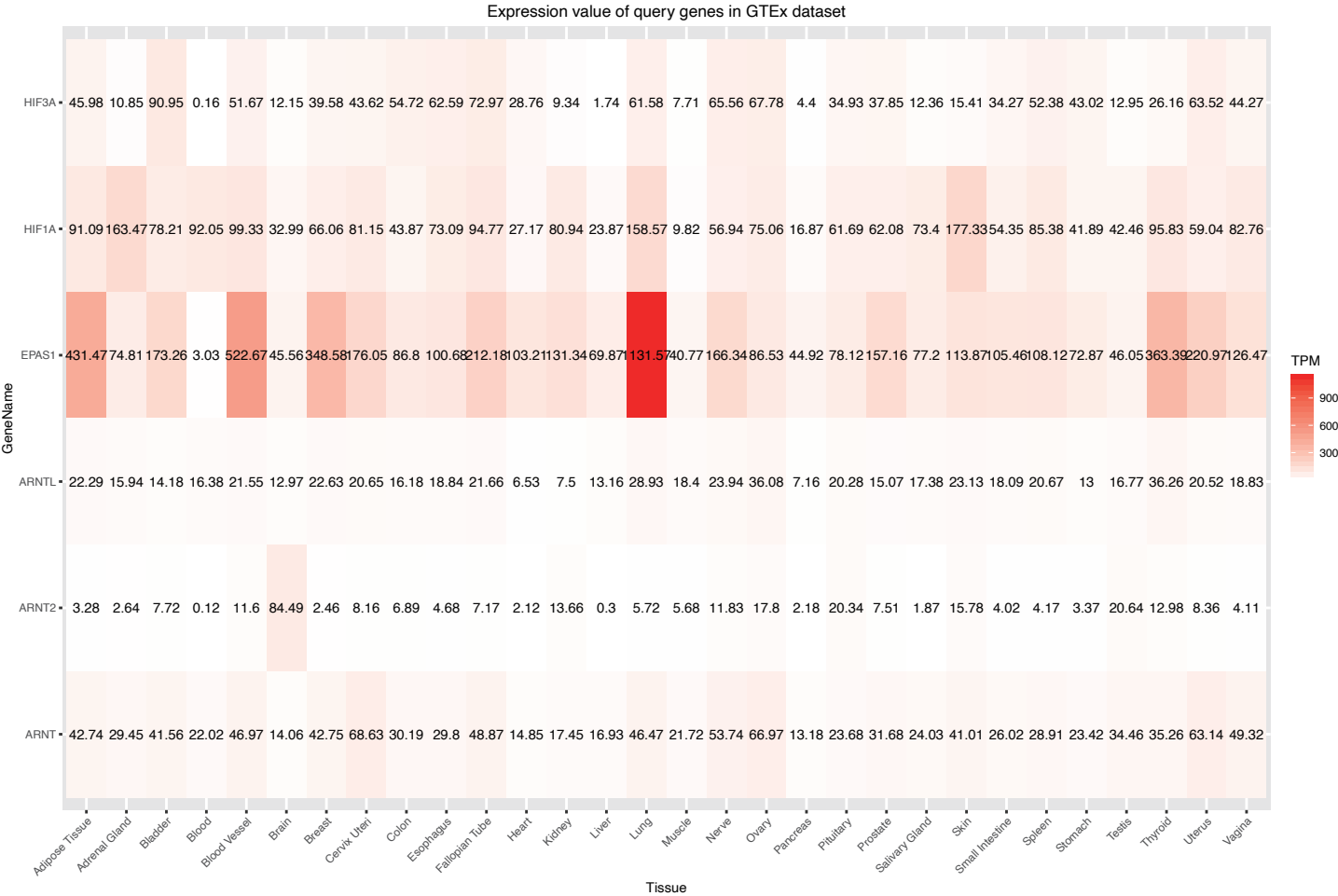

B

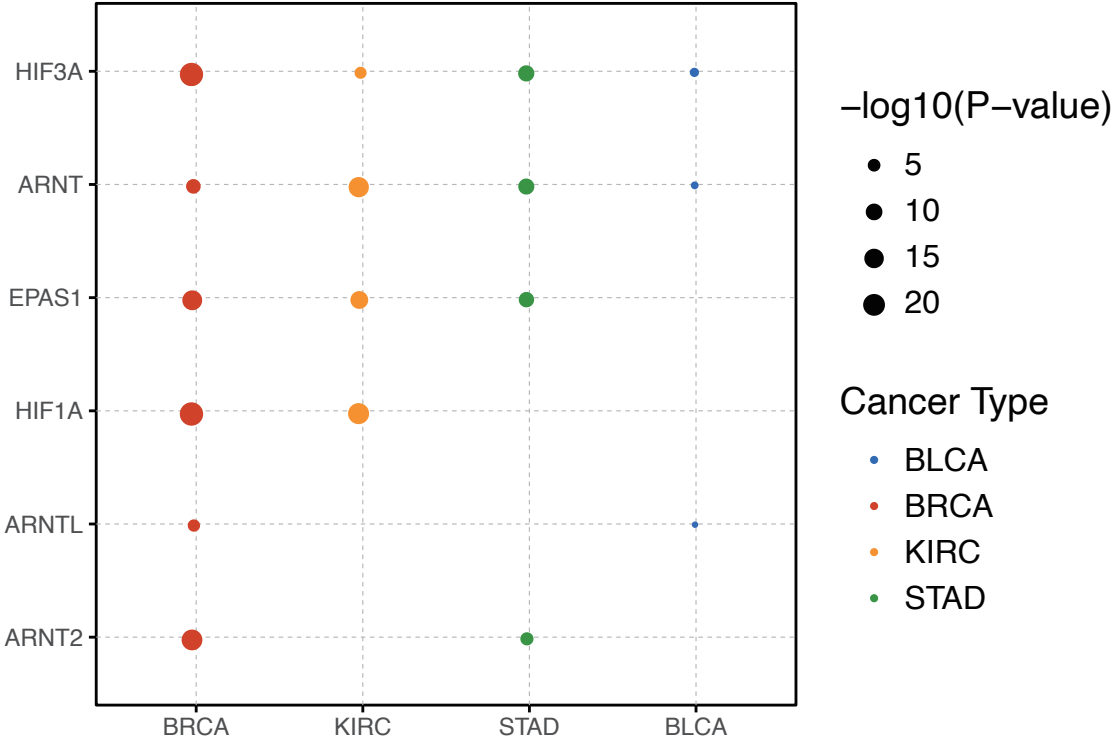

Supplement: Supplementary Materials — Figure S1: the expression of HIFs in normal tissues and cancer subtypes. (a) The mRNA expression of HIFs in normal tissues. (b) The expression of the HIF genes is different in cancer subtypes. Figure S2: copy number variation affects HIF gene expression. Copy number variant subtype of HIF gene in cancer. Hete Amp: heterozygous amplification; Hete Del: heterozygous deletion; Homo Amp: homozygous amplification; Homo Del: homozygous deletion; and no: no CNV. Figure S3: a review of HIF gene variants in human cancers. (a) Variant type, (b) variant classification, (c) single nucleotide variation, (d) variation per sample, (e) mutation classification, and (f) mutation of HIF gene between cancer types frequency. Figure S4: the HIF genes are widely associated with the hallmark cancer pathways in different cancer types. The pie plots represent the percentage of cancer samples that are correlated with HIF gene expression. [file 8873104.f1.zip › 8873104.f1/S Figure S1.pdf]

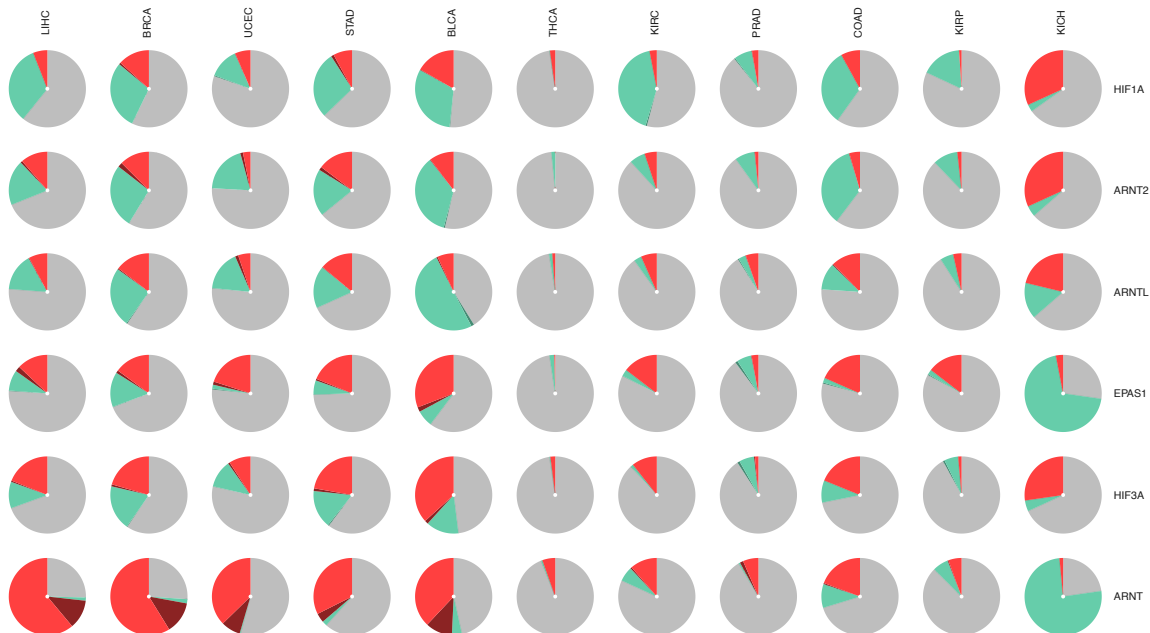

■ Hete Amp 
 ■ Homo Amp 
 ■ Hete Del 
 ■ Homo Del 
 ■ None

Supplement: Supplementary Materials — Figure S1: the expression of HIFs in normal tissues and cancer subtypes. (a) The mRNA expression of HIFs in normal tissues. (b) The expression of the HIF genes is different in cancer subtypes. Figure S2: copy number variation affects HIF gene expression. Copy number variant subtype of HIF gene in cancer. Hete Amp: heterozygous amplification; Hete Del: heterozygous deletion; Homo Amp: homozygous amplification; Homo Del: homozygous deletion; and no: no CNV. Figure S3: a review of HIF gene variants in human cancers. (a) Variant type, (b) variant classification, (c) single nucleotide variation, (d) variation per sample, (e) mutation classification, and (f) mutation of HIF gene between cancer types frequency. Figure S4: the HIF genes are widely associated with the hallmark cancer pathways in different cancer types. The pie plots represent the percentage of cancer samples that are correlated with HIF gene expression. [file 8873104.f1.zip › 8873104.f1/S Figure S2.pdf]

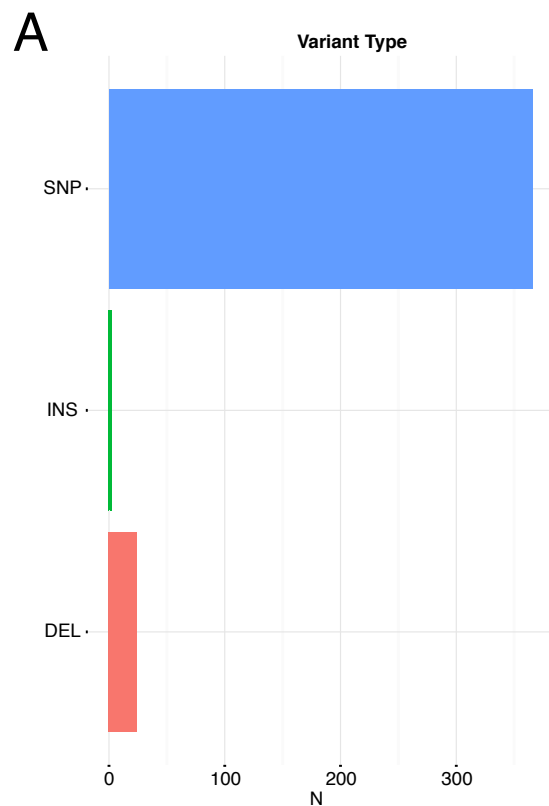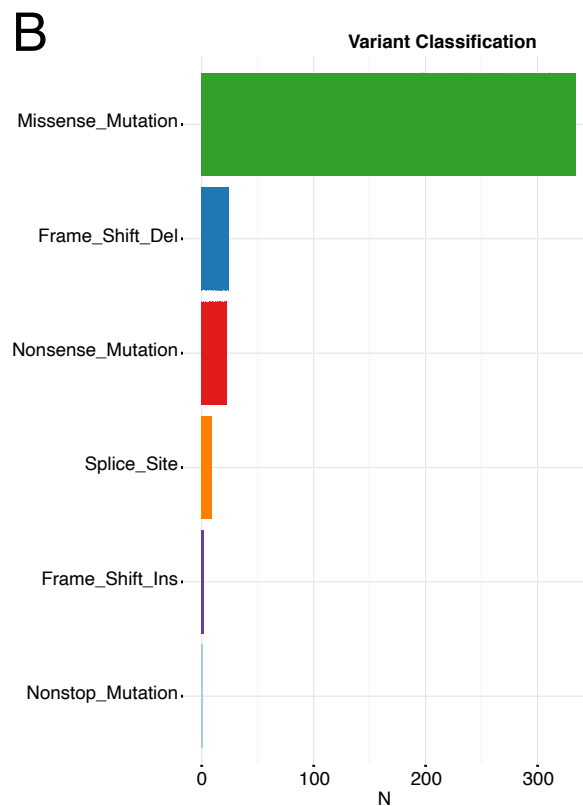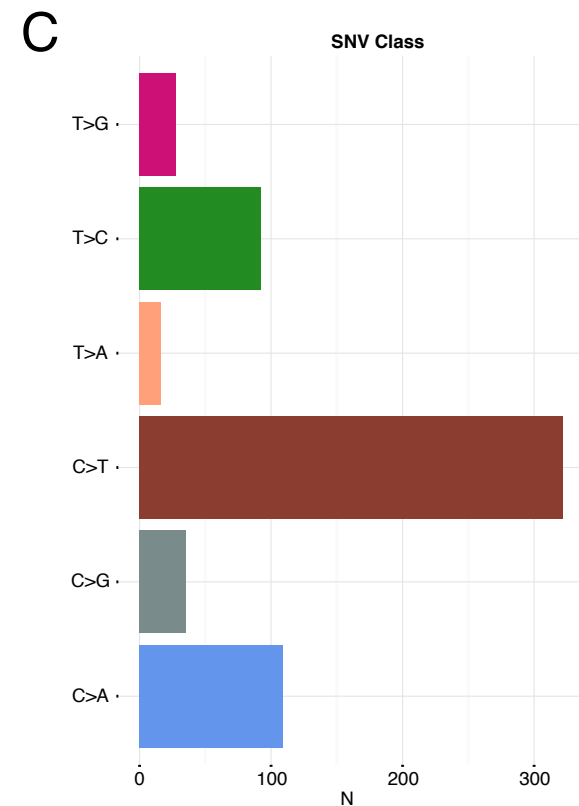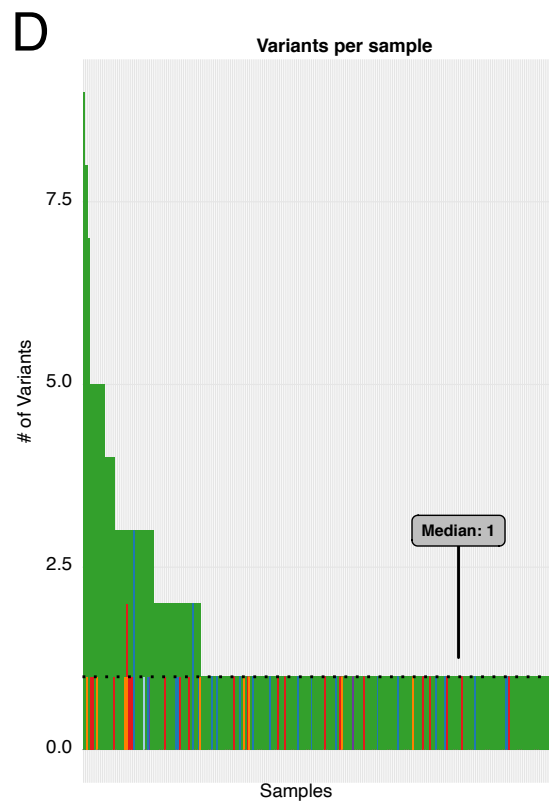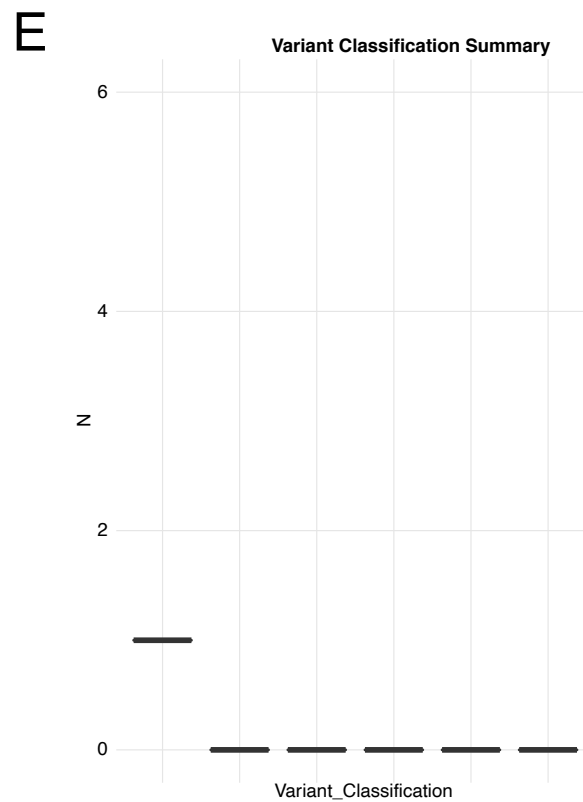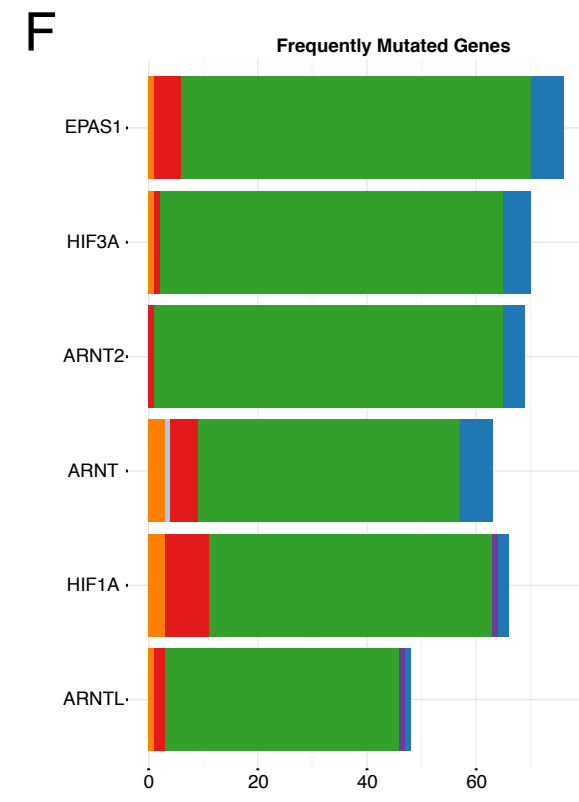

Supplement: Supplementary Materials — Figure S1: the expression of HIFs in normal tissues and cancer subtypes. (a) The mRNA expression of HIFs in normal tissues. (b) The expression of the HIF genes is different in cancer subtypes. Figure S2: copy number variation affects HIF gene expression. Copy number variant subtype of HIF gene in cancer. Hete Amp: heterozygous amplification; Hete Del: heterozygous deletion; Homo Amp: homozygous amplification; Homo Del: homozygous deletion; and no: no CNV. Figure S3: a review of HIF gene variants in human cancers. (a) Variant type, (b) variant classification, (c) single nucleotide variation, (d) variation per sample, (e) mutation classification, and (f) mutation of HIF gene between cancer types frequency. Figure S4: the HIF genes are widely associated with the hallmark cancer pathways in different cancer types. The pie plots represent the percentage of cancer samples that are correlated with HIF gene expression. [file 8873104.f1.zip › 8873104.f1/S Figure S3.pdf]

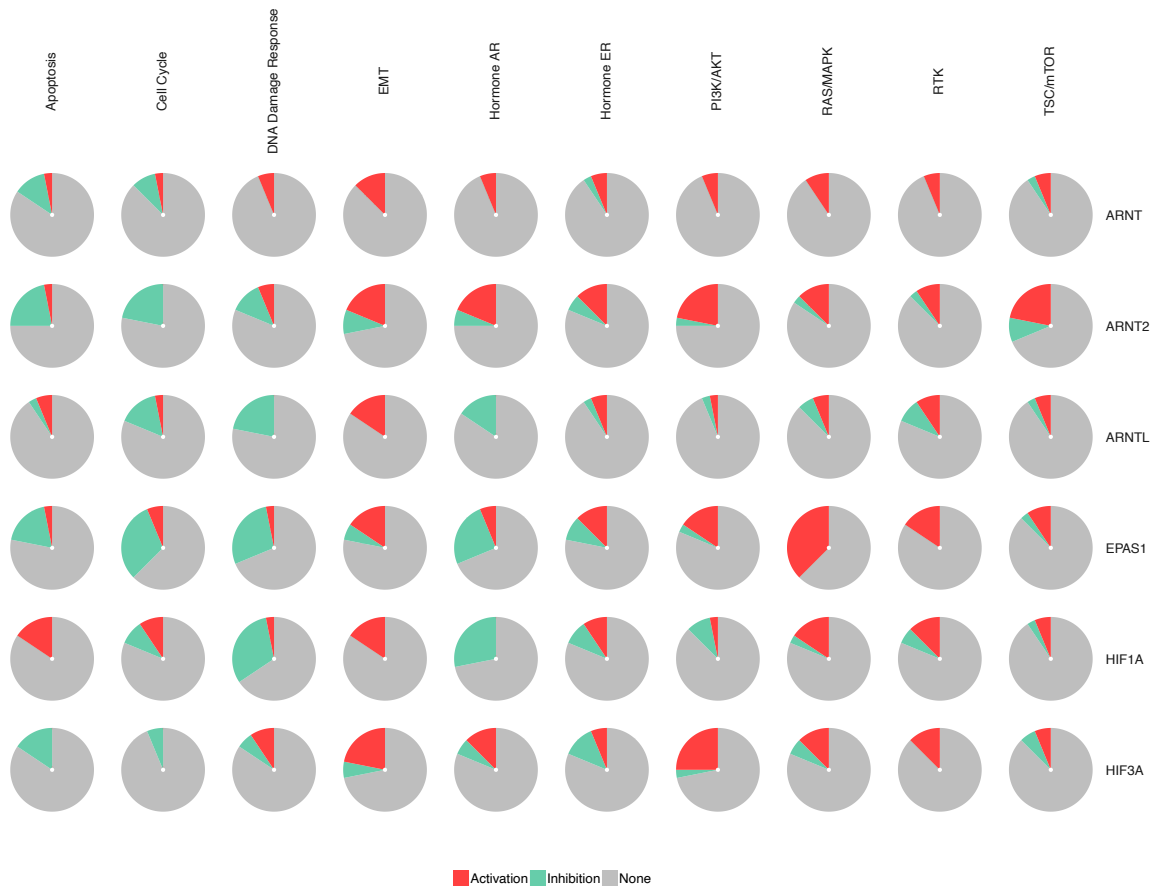

Supplement: Supplementary Materials — Figure S1: the expression of HIFs in normal tissues and cancer subtypes. (a) The mRNA expression of HIFs in normal tissues. (b) The expression of the HIF genes is different in cancer subtypes. Figure S2: copy number variation affects HIF gene expression. Copy number variant subtype of HIF gene in cancer. Hete Amp: heterozygous amplification; Hete Del: heterozygous deletion; Homo Amp: homozygous amplification; Homo Del: homozygous deletion; and no: no CNV. Figure S3: a review of HIF gene variants in human cancers. (a) Variant type, (b) variant classification, (c) single nucleotide variation, (d) variation per sample, (e) mutation classification, and (f) mutation of HIF gene between cancer types frequency. Figure S4: the HIF genes are widely associated with the hallmark cancer pathways in different cancer types. The pie plots represent the percentage of cancer samples that are correlated with HIF gene expression. [file 8873104.f1.zip › 8873104.f1/S Figure S4.pdf]
